# Posterior urethral hamartoma with hypospadias in a child: a case report and literature review

**DOI:** 10.3389/fped.2023.1195900

**Published:** 2023-08-22

**Authors:** Zipeng Hao, Chenghao Zhanghuang, Kun Zhang, Yu Hang, Fengming Ji, Bing Yan, Haoyu Tang

**Affiliations:** ^1^ Department of Urology, Kunming Children’s Hospital (Children’s Hospital Affiliated to Kunming Medical University), Kunming, China; ^2^ Yunnan Province Clinical Research Center for Children’s Health and Disease, Kunming Children's Hospital (Children's Hospital Affiliated to Kunming Medical University), Kunming, China; ^3^ Yunnan Key Laboratory of Children’s Major Disease Research, Kunming Children’s Hospital (Children’s Hospital Affiliated to Kunming Medical University), Kunming, China

**Keywords:** hamartoma, posterior urethra, hypospadias, children, treatment

## Abstract

**Background:**

Hamartoma is a mass formed by the proliferation and disorder of two or more kinds of cells inherent in normal organs or anatomical parts, which can occur in any part of the body. The most common hamartoma are kidney hamartoma, spleen hamartoma, liver hamartoma, and lung hamartoma. Urethral hamartoma is extremely rare in clinical practice.

**Case report:**

Combined with literature review, the diagnosis and treatment process of a child with posterior urethral hamartoma and hypospadias in our hospital were analyzed. The patient was cured after surgical treatment, the lesion was completely removed, the appearance was satisfactory, and there was no recurrence, urethral stricture, urethral fistula, and other complications. The pathological results of this case support the histological diagnosis of hamartoma, which provides reference for the clinical diagnosis and treatment of congenital malformation and tumor of urogenital in children.

**Conclusion:**

When a child has posterior urethral hamartoma, the symptoms may not be very typical, and it is often combined with urethral malformation. Therefore, it is necessary to perform careful physical examination combined with pathological examination to be able to make an accurate diagnosis. Under normal circumstances, the prognosis of urethral hamartoma is good. However, more cases are needed to be observed for verification, and a long-term effective follow-up after surgery is needed.

## Introduction

In clinical practice, hamartoma in the urethra is extremely rare, especially in the posterior urethra of children. X-ray imaging, color Doppler ultrasound, CT scan, MRI, and voiding cystourethrography (VCUG) are helpful in the diagnosis of this tumor. Most of the tumors are round solid masses, and generally tend to be benign. A surgical resection is the only effective method for the treatment of urethral hamartoma (1). Urethral cystoscopy before a resection is helpful to clarify the location, texture, and size of the tumor, as well as its relationship with the surrounding tissues. Combined with clinical manifestations, the diagnosis of benign tumors can be initially determined, and the final diagnosis depends on the histopathological examination. Here, we report the case of a patient with posterior urethral hamartoma that was recently treated in our department and share our experience in its diagnosis and treatment process.

## Case report

### Clinical data

**Figure 1:**
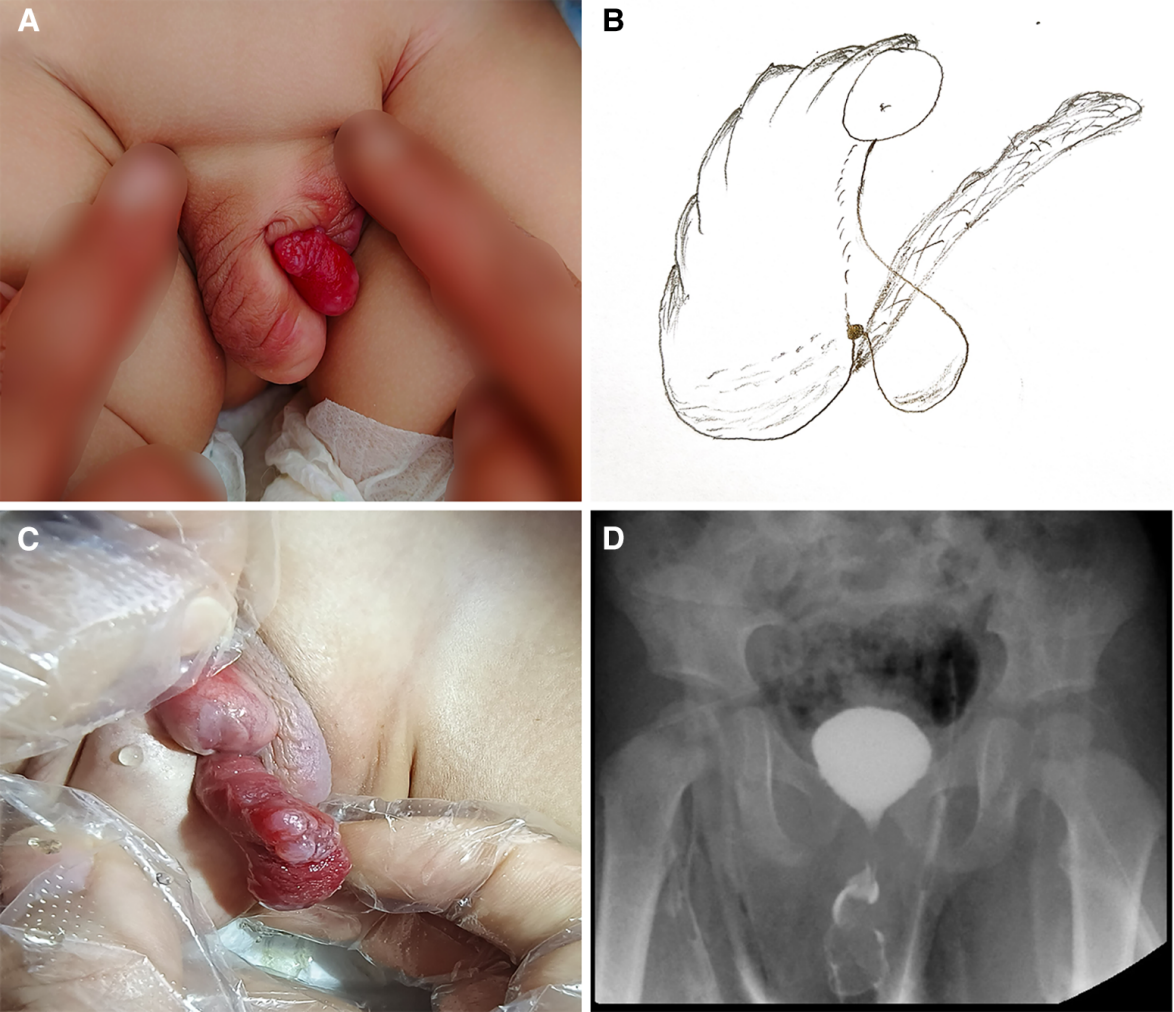
Preoperative findings: (**A**) general appearance of the perineum; (**B**) schematic diagram of the perineum; (**C**) location of perineal neoplasm; (**D**) cystic dilatation of the external orifice of the urethra in VCUG.

A boy aged 1 year and 5 months was admitted to the hospital because of ectopic urethral opening and perineal vegetation for more than 1 year. After birth, the parents found that the child has an abnormal appearance of the penis, with the penis bending downward, the urethra opening in the perineum, and a red polyp protruding from the perineum urethral orifice. As the child grows, the protrusion gradually increased and the deformity became more serious. The patient did not experience frequent urination, urination pain, urgency, or dysuria. Ultrasound examination in the outpatient department of our hospital showed that “there was no definite echo of the uterus and ovary in the pelvic cavity, and there was no obvious abnormality in the ultrasound images of the bilateral testes and epididymis,” and the chromosome examination showed “46, XY.” The outpatient was admitted to our department with “perineal vegetation unknown, perineal hypospadias.” The results of the physical examination conducted by the admission specialist were the following: the penis body of the child was short, the penis body and glans were poorly developed, the urethral opening was at the perineum of the ventral root of the penis, the penis was severely curved downward and was difficult to extend, and the foreskin was cap-like covering the dorsal side of the penis glans. The red polyp protruded from the perineal urethral orifice and was located approximately parallel to the penile body (Figures 1A,B). The protrusion was grossly cylindrical, about 2.0 cm in length and 0.6 cm in diameter, and resembled the neogranulation tissue. The surface was uneven, with ruddy mucosa without obvious skin coverage (Figure 1C). There was a bifid deformity in the bilateral scrotum; the bilateral scrotum touched the testes; the size was generally normal, without redness, swelling, tenderness, and thickening of the bottom of the right scrotum. There was also no abnormal mass. The auxiliary examination showed 46, XY chromosomes. The color Doppler ultrasound examination of the scrotum, adnexa, and uterus showed that there was no obvious abnormality in the bilateral testis and epididymis, and no exact echo of the uterus and ovary was detected in the pelvic cavity. The ultrasonography result showed a solid nodule in front of the penis (an inflammatory granuloma?). The VCUG showed that the urethra was short and the anterior part of the urethra was dilated in a cystic structure (Figure 1D), which was considered to be the perineal opening of the hypospadias. The MRI showed a patchy tissue in the perineum and urethra, with a size of 2.1 cm × 1.3 cm, which was approximately equal to the surrounding tissue. The enhanced scan showed an obvious uneven enhancement.

### Treatment methods

**Figure 2:**
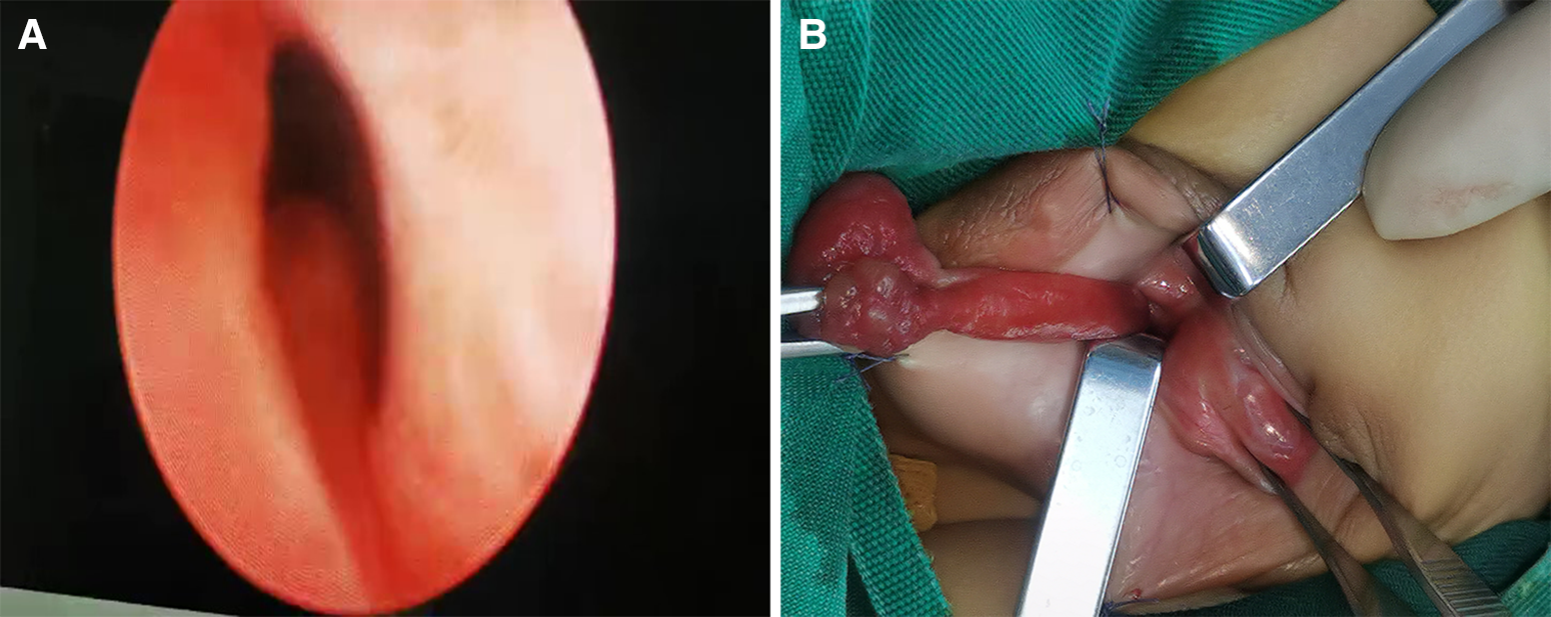
Intraoperative findings: (**A**) the root of the vegetation was located at the distal end of the verumontanum opening; (**B**) the root of the vegetation was pulled out during the operation.

The child underwent urethral vegetation resection and cystoscopy under general anesthesia. The urethral opening was found from the middle of the red vegetation protruding from the ventral penile body and perineum, and the cystoscope was inserted. The root of the abnormal vegetation in the perineum was located at the distal end of the verumontanum opening approximately 0.5 cm (Figure 2A). The vegetated part was wrapped by a little urethral mucosa. The urethra of the ventral part of the penis showed bright-red mucosa-like skin. The rest of the urethral mucosa and bladder neck mucosa were smooth without stenosis. After the cystoscope entered the bladder, the urine color was slightly mixed. The bilateral ureteral openings were in normal position without abnormal mass. Considering that the vegetation was large, the base was deep, and the urethral plate was damaged more after operation, a hypospadias surgery was not allowed for the time being. The child was placed in the supine position, and the root of the vegetation was pulled out as far as possible (Figure 2B). The complete vegetation was removed along the urethral plate skin of the vegetation root and was sent for examination. A careful hemostasis was performed, and the appearance of the urethral plate was formed by an intermittent suture with absorbable sutures. The results of intraoperative frozen pathology examination suggested that a benign tissue could be seen according to the specimen, the resection margin was clean and complete, and the root junctions are normal cells. The F10 double-lumen balloon catheter was indwelling and supported the urethra for 1 month after the operation.

## Results

**Figure 3:**
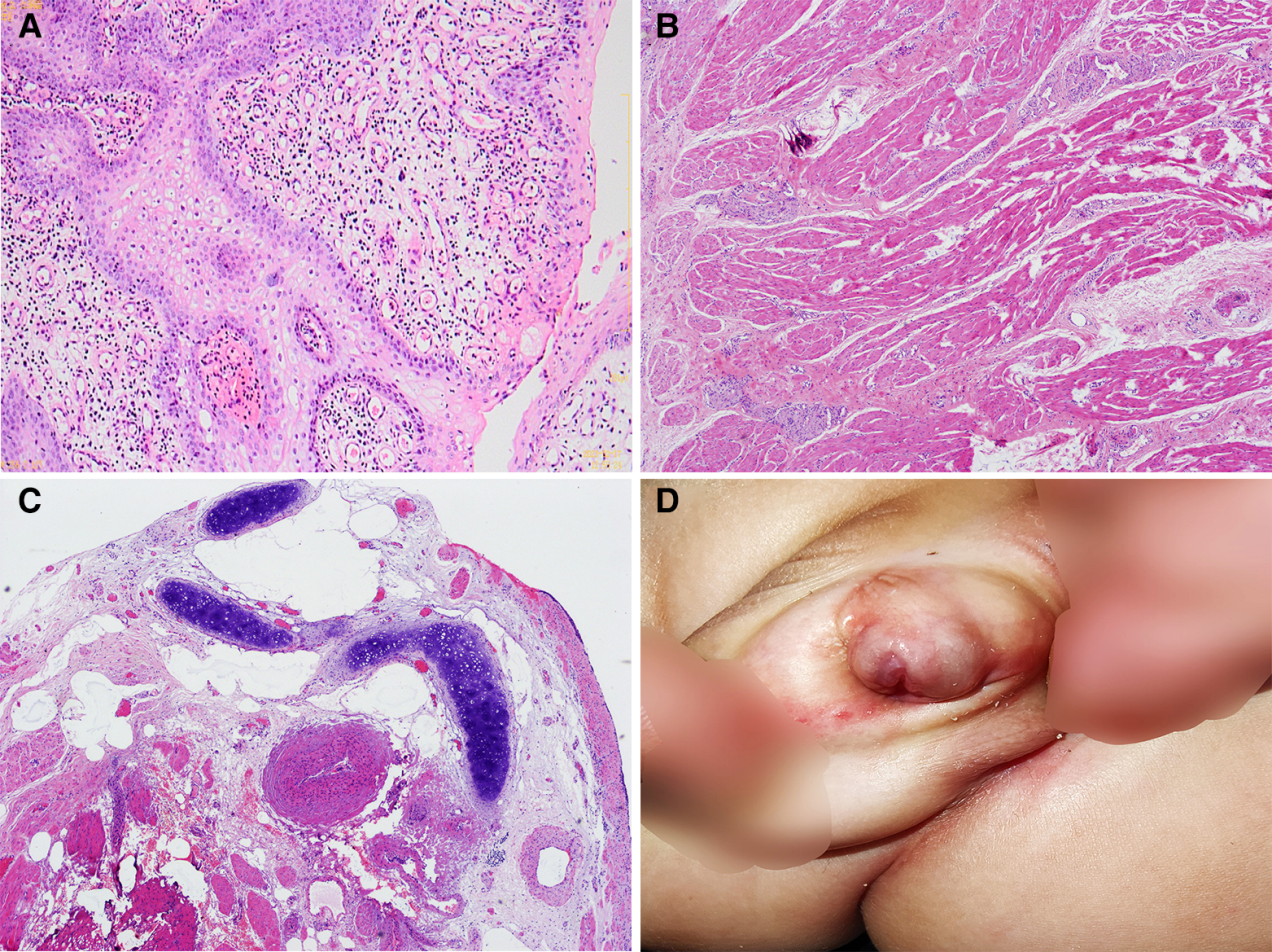
Intraoperative pathological examination and postoperative follow-up findings: (**A**) papillary hyperplasia of squamous epithelium with diffuse infiltration of chronic inflammatory cells (H&E, ×100); (**B**) hyperplasia of fibrous tissue, small blood vessels, smooth muscle, and nerve bundles (H&E, ×40); (**C**) coated with urothelium, cartilage islands, smooth muscle bundles, thick-walled blood vessels, and nerve bundles at the broken end (H&E, ×40); (**D**) findings at follow-up 1 month after surgery.

The operation was successful, and postoperative treatments such as prevention of infection, preventive hemostasis, nutritional rehydration, and dressing change were provided. The results of postoperative pathological examination showed that part of the examined tissues were polypoid hyperplasia, covered by the proliferative squamous epithelium, under which were proliferating fibers, small blood vessels, and smooth muscle, and diffuse infiltration of chronic inflammatory cells (Figures 3A,B). Some tissue was covered with urothelium, and cartilage islands, smooth muscle, blood vessels, and nerves were observed at the broken ends (Figure 3C). One lymph node was found locally, with lymphoid follicular hyperplasia, lymphatic sinus dilatation, and sinus histiocytosis. Combined with the above pathological results, the final diagnosis was hamartoma. The patient was discharged with a catheter, and the catheter was removed 2 weeks after discharge. The parents were informed after the operation. After the child recovered further, he was returned to the hospital for hypospadias repair.

The child was reexamined in the outpatient department of our hospital since early childhood, and through continuous physical examination comparison, it was known that the urethral vegetation continued to grow. We provided this case through relevant diagnosis and treatment and literature analysis of “urethral hamartoma.”

## Discussion

First reported by Albrecht in 1904, “hamartoma” is a tumor-like malformation caused by the wrong combination and arrangement of normal tissues in an organ during development (2). It is often a kind of abnormal development and growth of tissues after birth. This overgrown tissue still has the morphology of mature tissue and does not show the characteristics of tumor tissue, but sometimes it can develop into a tumor. Hamartoma is not a true tumor, and it grows slowly. It can grow with the development and growth of the body, but it automatically stops when it grows to a certain extent. It is more coordinated with the body and has a low malignant transformation rate (3). However, some studies have shown that hamartomas are true tumors [such as renal angiomyolipoma (4)], rather than abnormal combinations of normal tissues, and should be classified as mesenchymal tumors. The most common site of hamartoma is the kidney (5), followed by the lung (6), hypothalamus (7), and liver (8). It rarely occurs in the perineum (9, 10), and it is even rarer in the urethra (11), which has not been reported in China.

The main complaints of the child were the abnormal appearance of the penis and ectopic urethral opening. Since birth, the child was found by his parents to have ectopic urethral opening and a red polyp protruding from the perineal urethral opening, and the protrusion gradually increased with the growth of the child. Whether clinical diagnosis or imaging diagnosis, hamartoma has a high misdiagnosis rate (12). MRI is a useful diagnostic tool. Studies have shown that when a discrete pulmonary nodule shows neither fat nor calcification on CT scan, MRI can detect the very typical split-like structure in pulmonary hamartoma, which can provide a diagnostic basis (13). In this case, the color Doppler ultrasound tended to an “inflammatory granuloma,” the perineal sheet tissue was seen on MRI, and the signal was equal to the surrounding tissue. The enhanced scan showed an obvious uneven enhancement, and the outpatient physical examination tended to “repeated penile deformity” and “lymphangioma.” The causes of misdiagnosis were summarized as follows: first, the clinical cases were rare; second, the appearance of hamartomas does not have obvious characteristics such as cysts, polyps, hemangiomas, and other lesions (14). Throughout the entire perineal physical examination of the patient, it is worth mentioning that this case was based on the deformity of the penis and urethral orifice (perineal hypospadias). Early chromosome examination can further exclude the possibility of abnormal sex development. In this case, both the preoperative VCUG examination and intraoperative cystoscopy exploration showed that the perineal opening of hypospadias was blocked by hamartoma tissue, resulting in a fissure and stenosis of the external urethral orifice, increasing urethral resistance, shortening and narrowing of the normal posterior urethra, and dilatation of the local urethra in a cystic structure. In addition, the root of the neoplasm is suspected to gradually extend to the proximal end of the posterior urethra and approach the verumontanum, which increases the difficulty of a resection during operation.

A complete surgical resection is the only effective treatment for posterior urethral hamartoma. In this case, the mass was completely removed during operation, and the results of the postoperative pathological examination supported the histological diagnosis of hamartoma. Histologically, the main components of hamartoma include mesenchymal tissues such as cartilage, fibrous tissue, adipose tissue, nerve, and blood vessels (15). The proportion of each component is different, and it is usually named after the dominant component, which can be divided into chondroid hamartoma, chondromesenchymal hamartoma, angioid hamartoma, epithelioid hamartoma, etc. (16). The prognosis of hamartoma is good without postoperative radiotherapy or chemotherapy. Up to now, the child has no recurrence, no urethral stricture, and no urinary incontinence and other related symptoms, which makes a good condition for the later repair of hypospadias. In addition, it is worth noting that due to the short follow-up time of this case, a long-term follow-up should be strengthened in combination with the continued diagnosis and treatment of hypospadias in the future. This case report is extremely rare in clinical practice, which can further deepen the understanding of urogenital malformations and tumors in children, provide a certain theoretical basis for clinical diagnosis, and reduce the misdiagnosis rate.

## Conclusion

Posterior urethral hamartoma complicated with hypospadias is rare in children, and more clinical practice studies are needed. Before surgery, it is necessary to perform conventional series of examinations such as x-ray imaging, color Doppler ultrasound, CT scan, MRI, and VCUG. Before a formal resection, urethrocystoscopy is needed to further clarify the nature and location of the tumor and the condition of the urethra. During the resection, attention should be paid to reduce the damage of urethral mucosa, so as to minimize the influence on the subsequent urethroplasty. Therefore, in patients with urethral hamartoma, more cases to be observed and a longer-term effective follow-up are needed for verification.

## Data Availability

The original contributions presented in the study are included in the article, further inquiries can be directed to the corresponding authors.
